# Aluminum Poisoning with Emphasis on Its Mechanism and Treatment of Intoxication

**DOI:** 10.1155/2022/1480553

**Published:** 2022-01-11

**Authors:** Mehrdad Rafati Rahimzadeh, Mehravar Rafati Rahimzadeh, Sohrab Kazemi, Roghayeh Jafarian Amiri, Marzieh Pirzadeh, Ali Akbar Moghadamnia

**Affiliations:** ^1^Department of Nursing, Babol University of Medical Sciences, Babol, Iran; ^2^Department of Medical Physics, Kashan University of Medical Sciences, Babol, Iran; ^3^Cellular and Molecular Biology Research Center, Health Research Institute, Babol University of Medical Sciences, Babol, Iran; ^4^Student Research Committee, Health Research Center, Babol University of Medical Sciences, Babol, Iran; ^5^Department of Pharmacology, Babol University of Medical Sciences, Babol, Iran

## Abstract

Aluminum poisoning has been reported in some parts of the world. It is one of the global health problems that affect many organs. Aluminum is widely used daily by humans and industries. Residues of aluminum compounds can be found in drinking water, food, air, medicine, deodorants, cosmetics, packaging, many appliances and equipment, buildings, transportation industries, and aerospace engineering. Exposure to high levels of aluminum compounds leads to aluminum poisoning. Aluminum poisoning has complex and multidimensional effects, such as disruption or inhibition of enzymes activities, changing protein synthesis, nucleic acid function, and cell membrane permeability, preventing DNA repair, altering the stability of DNA organization, inhibition of the protein phosphatase 2A (PP2A) activity, increasing reactive oxygen species (ROS) production, inducing oxidative stress, decreasing activity of antioxidant enzymes, altering cellular iron homeostasis, and changing NF-kB, p53, and JNK pathway leading to apoptosis. Aluminum poisoning can affect blood content, musculoskeletal system, kidney, liver, and respiratory and nervous system, and the extent of poisoning can be diagnosed by assaying aluminum compounds in blood, urine, hair, nails, and sweat. Chelator agents such as deferoxamine (DFO) are used in the case of aluminum poisoning. Besides, combination therapies are recommended.

## 1. Introduction

Access to various chemicals has caused many cases of poisoning in the last decades [1, 2]. People are deliberately or accidentally poisoned by misuse or overdose of drugs and chemicals [3, 4]. Heavy metals are important examples among these agents. These materials are released from natural sources or industrial wastes and can threaten human health [5].

One of these agents is aluminum (Al), which is a silvery-white, soft, malleable, nonmagnetic, and ductile metal [6, 7]. Aluminum forms about 8% of the Earth's crust and is the third most abundant element after oxygen and silicon [8, 9]. The atomic number and atomic mass number of aluminum are 13 and 26.98, respectively. It has a melting point of 660.32°C and a boiling point of 2518°C [6, 10]. It has several isotopes. ^27^Al has an abundance of over 99.9% and is a stable isotope, and ^26^Al is a radioactive isotope with a half-life of 7.2 × 105 years. Except for ^26^Al, all of the radioisotopes have a half-life of less than 7 minutes [11].

Aluminum is combined with more than 270 different minerals. Aluminum is combined with oxygen, fluorine, silicon, sulfur, and other forms. This does not happen in elemental status [10]. The original aluminum ore, a mixture of hydrated aluminum oxide (Al_2_O_3_. *x*H_2_O) and hydrated iron oxide (Fe_2_O_3_. *x*H_2_O), is known as bauxite. In addition, cryolite (Na_3_AlF_6_) is another important mineral in the production of aluminum metal. There are 3 main classes in the aluminum alloy: (1) pure aluminum, (2) heat treatable aluminum alloy, and (3) nonheat treatable aluminum alloy (Table 1) [11, 12]. Aluminum and its alloys are used in numerous ways in daily life of humans and industries. Aluminum, a corrosion-resistant metal, has light weight and density, high electrical and thermal conductivity, and high ductility and is easily deformable. For this reason, it is used in aerospace industries, transportation industries, packaging and food industries, building and construction, electrical industries, a wide range of home household and appliances, machinery and equipment, and monetary currency like aluminum coins [11, 13].

## 2. Exposure Ways to Aluminum

The majority of people ignore the exposure to aluminum in their daily activities. Perhaps our understanding of “exposed to aluminum” is related to the presence of aluminum in food sources. This point is correct in some ways, but the other points should be considered remarkably. The main source of oral intake of aluminum is food, which accounts for approximately 95% of the daily intake of food, and drinking water consists of 1-2%. These usually provide 4,000–9,000 micrograms intake per day [14]. Also, using antacids may increase oral intake up to 5,000,000 micrograms. Daily entry of aluminum into the body through respiration may be 4–20 micrograms. This number is increased up to 25,000 micrograms by inhalation in industrial places, and finally, exposure to deodorants containing aluminum compounds may increase entrance of this metal into the body up to 50,000–75000 micrograms per day [14, 15].

Aluminum-based adjuvants are used in the immunotherapy of allergic conditions. Almost 75% of all adjuvant-based treatments include aluminum salts. The amount of aluminum salts in the vaccine depends on the manufacturer; for example, low-dose diphtheria/tetanus vaccine contains 170 micrograms per dose, or high-dose haemophilus influenza type b vaccine contains 850 micrograms of the salts in each dose [16, 17]. Aluminum exposure can also occur intravenously, such as the presence of aluminum in dialysis solution and total parenteral nutrition solutions. Because 110 liters of dialysis solution is used three times a week and total parenteral nutrition in infants and children is based on 0.1/kg/day and in adults 2 liters per day, it causes the accumulation of aluminum leading to poisoning [15].

## 3. Pharmacokinetics and Toxic Kinetics

### 3.1. Absorption

Aluminum is a ubiquitous element, and people are easily exposed to it. One way of aluminum exposure is food consumption, which is absorbed through the mucosa of the small intestine [18]. Aluminum is then entered into the bloodstream in two ways, passive diffusion and active transport or via transferrin-mediated mechanisms [19]. Aluminum salts, such as aluminum chlorohydrate (ACH), are active components of antiperspirants and deodorants. Their mechanism of action is deposition in the eccrine sweat glands and producing insoluble aluminum hydroxide. This causes loosening of sweat plaques and blocking sweat secretion [20, 21]. Aluminum respiratory uptake and pulmonary absorption are about 1.5 to 2% in patients exposed to aluminum containing fumes [19].

### 3.2. Distribution

Aluminum serum concentration is almost equal to whole blood aluminum levels in a normal situation. Almost 90% of plasma aluminum is bound to transferrin, between 7 to 8% with citrate, and less than 1% binds to phosphate and hydroxide. Approximately 60% of the body aluminum is stored in the bones, 25% in the lung, 10% in the muscle, 3% in the liver, and 1% in the brain [19, 22]. Inside the cells, aluminum is stored in the lysosomes of brain neurons, liver (except the Kupffer cells), spleen, myocytes of the heart, mesenchymal glomerular cells, epithelial cells of the kidneys, and mitochondria of osteoblasts [19].

### 3.3. Excretion

More than 95% of aluminum is excreted through the kidney. In fact, the main route of systemic aluminum elimination is through the kidneys, while aluminum excretion through bile is about 2%. Healthy subjects under normal situations are able to excrete all absorbed aluminum. When people are exposed to high levels of aluminum, for example, in the case of total parenteral nutrition, aluminum cannot be excreted in a first-order kinetic profile and some parts of the absorbed aluminum may be accumulated. One of the reasons for this accumulation is the protein binding that limits the ability of aluminum ultrafiltration. Aluminum clearance depends on frequency, type, and route of exposure. Although most of the absorbed aluminum is excreted in the first week after exposure, it is estimated that the excretion process may last between a few hours, a few days, and even a few years [22, 23].

## 4. Mechanism of Aluminum Poisoning

Aluminum is the third most common and ubiquitous element in the world. It is naturally found in air, soil, and water. In addition, this element has gained GRAS (generally recognized as safe) designation by FDA (Food and Drug Association), so it is possible to be added illegally not only to drinking water resources but also to many foods and medicines. Recent studies on environmental poisoning have shown that aluminum can be a major threat to humans, animals, and plants [24–26]. Overdose of aluminum provides oxidative stress in the brain, liver, and kidney. It is possible to enhance free radicals and change antioxidant capacity of the enzymes. Any exposure to aluminum may disrupt or inhibit several enzymes and change protein synthesis, nucleic acid function, and cell membrane permeability. It can also affect the plasma levels of triglycerides and their metabolism in the body [27–29].

Aluminum is a prooxidant and may lead to biological oxidation both *in vitro* and *in vivo*. Aluminum increases lipid peroxidation (LPO) with reduction in glutathione (GSH) content, glutathione peroxidase (GSH-Px), glutathione S-transferase (GST), and catalase (CAT) activities in renal tissue [30, 31]. The accumulation of aluminum in renal tissue leads to the degeneration of renal-tubular cells, affects cellular metabolism, and stimulates oxidative stress, thus leading to changing p-aminohippuric acid transport and reabsorption of phosphate in kidney tubules and imbalances in sodium and water and finally causing nephrotoxicity [30].

Sodium-potassium ATPase is the main protein complex in the family of P-type ATPase in all living organisms. It plays a role in maintaining the gradient outside and inside the cell and its homeostasis. Aluminum inhibits the action of Na+/K + ATPase both *in vitro* and *in vivo* [32, 33]. Aluminum produces reactive oxygen species (ROS), leading to lipid peroxidation and oxidative damage to proteins and DNA. It causes destructive changes in the liver cells and degeneration in the rough endoplasmic reticulum (RER) that is associated with a decrease in protein synthesis in the liver and may also lead to changes in cellular calcium in the cell [34, 35]. Under aluminum toxicity conditions, swelling occurs in most mitochondria of the liver cells, and changes in the permeability of the mitochondria membrane can exacerbate redox conditions of the mitochondrial thiol group, which may affect the amount of free calcium in the cell. This damage to liver parenchymal cells increases serum alanine aminotransferase (ALT) activity and malondialdehyde (MDA) up to 4 times and enhances proinflammatory cytokines such as TNF-*α* to 7 times than the normal amounts [35].

Due to the similarity of ionic radii of Al^3+^ and Fe^3+^, there is the possibility of Al^3+^ appearing in Fe^3+^ sites. Therefore, aluminum is bound to Fe^3+^ transporting protein. This mechanism may decrease in binding of Fe^2+^, augment free intracellular Fe^2+^, and result in the peroxidation of membrane lipids leading to membrane damage [35]. On the other hand, endoplasmic reticulum function helps in the release of Ca^2+^ activators and signals discharge of endoplasmic reticulum Ca^2+^ stores. The release of Ca^2+^ into the cytoplasm, in addition to the depletion of Ca^2+^ stores, affects organic cellular operations such as regulation of gene expression, cell growth, and cell apoptosis [34] (Table 1).

Experimental studies suggest the neurotoxic effects of aluminum compounds due to the generation of ROS and free radicals [36]. Aluminum compounds affect different organelles such as mitochondria, lysosomes, and nuclei [37]. Several studies have reported that exposure to aluminum increases ROS production, which results in electron leakage, augmented mitochondrial activity and improved electron-chain activity. With ROS damage to mitochondria as its target organelle, the structure and function of the cell change. This will provide a series of events in cells, such as alternation in mitochondrial function, which reduces the mitochondrial membrane potential (MMP) that is vital for living cells, including neural cells. Reducing MMP inhibits the activity of the enzymes in the electron transport chain, decreases ATP, and increases electron leakage. Following this, depolarization of the nerve cell membrane and influx of calcium ions occur. In general, ROS and oxidative stress disturb cellular signaling and neurotransmission and result in cytotoxicity. The consequence of this process is neural damage, neurodegeneration, and death of neural cells [38–40].

Aluminum accumulation in the bone marrow results in osteomalacia. A reduction in the number of osteoblasts and osteoclasts and reduced bone marrow remodeling are associated with increased aluminum levels in the body. Aluminum can also inhibit hemoglobin synthesis in the bone marrow that is probably a result of protein synthesis inhibition by aluminum. Reduced hemoglobin synthesis can result in microcytic hypochromic anemia [41]. Aluminum deposition in rat lungs caused polymorphonuclear leukocytes (PMN) accumulation and a dose-dependent inflammatory state. Besides, a reduction in alveolar lavageable macrophages as well as type II cell hyperplasia has been observed, followed by aluminum entrance to the lungs [42] (Table 1).

Brain gene transcription in the presence of aluminum leads to a deficiency of genetic information, immunological signals, and destruction of DNA in the mammalian brain. Aluminum salts may bind to DNA and RNA and prevent their formation. This is achieved by single- and double-strand break of DNA in different phases of the cell cycle. Exposure to aluminum compounds can also change the stability of the DNA structure and prevent DNA repair [43, 44]. When aluminum loading occurs, aluminum gradually accumulates in human neurons, which inhibits the protein phosphatase 2A (PP2A) activity and/or hyperphosphorylation of tau. Inhibition of PP2A activity can lead to protein hyperphosphorylation in neurons such as tau protein, neurofilament protein, and other proteins. Hyperphosphorylation is a consequence of an imbalance between the kinases that are added to the phosphates and the phosphatases that are taken from the proteins. The recently synthesized hyperphosphorylated proteins accumulate in the neurons, making them vulnerable [45].

Some animal experiments have shown that the presence of aluminum in the brain increases iron content. The structure of the iron regulatory elements (IREs) is composed of a number of iron proteins, including ferritin and transferrin receptors in mRNAs. Two proteins bound to IRE, IRP-1, and IRP-2 act as iron sensors. When iron is used in the cell, IRP-1 combines with the iron-sulfur branch and becomes a form that cannot connect to the IRE, while IRP-2 rapidly degrades. Studies have shown that the IRP-2 may be stabilized by aluminum preventing its degradation, which leads to an increase in the biosynthesis of the transferrin receptor and the prevention of ferritin production. Low concentrations of aluminum increase the removal of nontransferrin bound iron and iron bound to transferrin in brain cells in humans. In addition, the amount of iron bound to ferritin decreases. Increasing iron in the cytoplasmic pool may indicate increased oxidative stress. On the other hand, high iron content did not change the protective enzymes of the cell, such as superoxide dismutase, glutathione reductase, glutathione peroxidase, and catalase. In contrast, when aluminum was administrated orally, lipid peroxidation levels have notably increased, and the activity of superoxide dismutase, glutathione peroxidase, and catalase has decreased. Studies have suggested that even small amounts of aluminum in the brain may alter iron homeostasis in the brain, causing neurodegenerative disorders [46, 47].

Aluminum can induce corticoneuronal apoptosis, and it is possible that SAPK/JNK (stress-activated protein kinase or c-jun N-terminal kinase) signal transduction pathway plays an important role in apoptosis in this case [48]. Moreover, the transcription factor NF-kB is operated by many signaling events and is implicated in numerous regulatory pathways. NF-kB family are responsible for regulating lots of genes, as well as proinflammatory cytokines, immune receptors, cell adhesion molecules, chemokines, and microRNAs. The signal transductions mentioned are involved in a wide range of biological processes, such as cell growth, differentiation, inherent immunity, inflammation, tumor cell growth, ROS production, and apoptosis; some of them will cause inflammatory neurodegeneration and consequently impair the normal functioning of the central nervous system in humans [49, 50] (Table 1).

Other studies with different pathways and mechanisms suggest the induction of neurotoxicity by aluminum. Bcl-2 (B-cell lymphoma 2) and BAX (B-cell lymphoma-associated X) genes encode the production of proteins that control cell death Normally, the expression of Bcl-2 prevents apoptosis, while BAX induces apoptosis. One of the possible contributing factors, which is p53 (p53 plays a role in regulating cell cycle, DNA repair, and apoptosis), controls Bcl2/BAX and also other main genes related to apoptosis. The BAX is an important mediator of the internal pathway of the mitochondria, which leads to caspase 3 activation and apoptosis. p53 directly transactivates BAX gene expression and enhances BAX production. Observations and studies indicated that aluminum compounds decrease Bcl-2 and increase BAX expression with an increase in p53 expression, resulting in apoptosis, which may be controlled by the p53 pathway. Of course, this requires more studies [51, 52] (Table 1).

## 5. Pathophysiology and Clinical Manifestation

**Figure 1:**
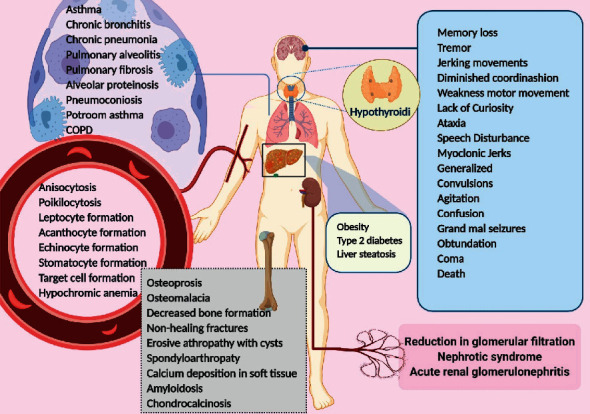
Pathophysiology and clinical manifestations of aluminum toxicity. Aluminum toxicity affects different body organs, including brain, parathyroid gland, kidney, lungs, liver, bones, and bone marrow, leading to various clinical manifestations. Aluminum effect on bone marrow leads to the formation of abnormal red blood cells besides its effect parathyroid gland and on musculoskeletal system is represented by abnormalities like osteoporosis and osteomalacia. Liver stenosis and nephrotic syndrome are other important manifestations of aluminum toxicity. Brain and respiratory system can also be severely damaged, followed by aluminum poisoning. Memory loss, tremor, jerk, and death are important manifestations of brain injury. Lung injury can be represented by different clinical manifestations such as asthma and chronic bronchitis (created with BioRender.com).

Aluminum affects the parathyroid hormone-calcium axis and acts various mechanisms in the parathyroid glands. Aluminum accumulates in the parathyroid glands, reduces the parathyroid response to hypocalcemia, and prevents the release and synthesis of parathyroid hormone (PTH). This action reduces serum calcium and prevents bone mineralization [53]. The most important changes in aluminum poisoning occur in relation to the musculoskeletal system in patients who are at the end stage of kidney disease and require dialysis. They receive dialysate fluid, as well as aluminum hydroxide taken as a phosphate binder to prevent hyperphosphatemia. Another group of patients with aluminum-induced bone disease is seen in other patients with a peptic ulcer that receives high-dose antacids. A wide range of diseases, including osteoporosis, osteomalacia, decreased bone formation, and progress of nonhealing fractures, occur due to the aluminum accumulation in patients undergoing dialysis. Aluminum accumulates in the mineralization front of the bone surface, where new type 1 collagen synthesis osteoblasts are located, which disrupts calcification, leading to osteomalacia, hypercalcemia, and hypercalciuria. One potential consequence of substituting aluminum for calcium on the bone surface would be hungry bone syndrome. Another consequence of this accumulation in the bone is the inhibition of the enzyme 25-hydroxyvitamin D-1 alpha-hydroxylase, which converts 25-hydroxy D to 1 alpha, 25-dihydroxyvitamin D, reducing the active metabolic form of vitamin D [54]. In patients with long-term dialysis, erosive arthropathy with cysts, spondyloarthropathy with calcium deposition in soft tissues, beta2-microglobulin amyloidosis, and chondrocalcinosis can be observed [55] (Figure 1) (Table 2).

Aluminum affects human erythropoiesis as well as adult erythrocytes. Aluminum changes the morphology of red blood cells, which is seen as anisocytosis and poikilocytosis in blood smear, leptocytes, acanthocytes, echinocytes, stomatocytes, and target cells (Figure 1) [56].

Hepatocytes play a role as liver function units and, with the help of a complex of metabolic networks, maintain homeostasis in the human body. Different toxins in the environment lead to liver dysfunction. Mitochondrial metabolism and aerobic respiration are the main functions of hepatocytes. The mitochondria in hepatocytes maintain the homeostasis of carbohydrates and lipids, biosynthesis reactions, and amino acid metabolism. Aluminum leads to iron depletion in the mitochondria, oxidative stress, and anaerobic respiration. This situation leads to an increase in the synthesis of the transferrin receptor and the suppression of ferritin production. Aluminum intervention with numerous iron-dependent enzymes in the tricarboxylic acid (TCA) cycle and the electron transport chain (ETC) leads to a reduction in the production of ATP by mitochondria through oxidative phosphorylation. Mitochondrial dysfunction in aluminum poisoning results in an increase in anaerobic conditions and lipid accumulation. This accumulation of lipids within the cell is because of increased lipogenesis and decreased oxidation of beta-fatty acids, which causes the main change and alternation in alpha-ketoglutarate (KG) and homeostasis. Ideally, KG is used to suppress ROS in aluminum exposed cells leading to succinate accumulation and stabilization of hypoxia-induced factor1-*α*. Also, the activity of KG to counteract oxidative stress leads to a reduction in the biosynthesis of L-carnitine and at the same time reduces the oxidation of fatty acids. Lipid accumulation is enhanced by aluminum-induced changes in the levels of KG, succinate, Nicotinamide Adenine Dinucleotide Phosphate (NADPH), and L-carnitine. These conditions provide various liver diseases [57]. In addition, Zhu et al. have shown that an increase in aluminum concentrations suppresses the cytochrome P450(CYP450), cytochromeB5(B5), microsomal protein, cytochrome C reductase (CR), aminopyrine N-demethylase (AND), erythromycin N-demethylase (ERND), and aniline-4-hydroxylase (AH), playing a major role in the anabolism of endogenous compounds and biotransformation of many exogenous compounds [58]. These events have a major impact on liver diseases, including obesity, type 2 diabetes, and liver steatosis (Figure 1). In fact, these disorders occur with a significant reduction in aerobic respiration, mitochondrial lipid oxidation disturbance, increased lipid, and VLDL secretion [57, 59].

The accumulation of aluminum in the kidney causes glomerular filtration failure and damages tubular kidney cells, leading to nephrotoxicity. Almost all aluminum in plasma is integrated with high molecular mass (HMM) or low molecular mass (LMM). Meanwhile, 80 to 90 percent of aluminum is bound to protein (mostly transferrin) and is therefore unfilterable, and the rest is bound to lower molecular masses such as citrate, which is the most important. In patients with renal failure, the plasma concentration of aluminum increases strongly due to the increased concentration of plasma phosphate, which leads to an increase in the low molecule binding. It is thought that, in high concentrations of aluminum, filtration could be reduced due to the formation of insoluble complexes. However, it can be stated that, in the case of elevated plasma concentrations of aluminum, filtration is strongly related to the nature of onions, which aluminum is bound to. That is why citrate administration in the case of aluminum poisoning could be of great importance that it helps aluminum filtration. Furthermore, fractional aluminum reabsorption is decreased in the case of elevated plasma aluminum conditions. Changes in serum levels of urea, creatinine, and uric acid are seen in these patients, which is due to impaired renal function. In addition, aluminum can lead to nephrotic syndrome or acute renal glomerulonephritis, which causes proteins to be excreted in the urine (Figure 1) [60, 61].

All people, especially workers exposed to aluminum compounds, suffer from respiratory diseases, and deficiency of pulmonary function tests has been observed in them. These tests consist of forced expiratory volume in the 1st second (FEV1), forced vital capacity (FVC), ratio of these two factors in percentage ( (FEV1/FVC)%), and forced midexpiratory flow rate (FEF%) that implies a wide range of respiratory diseases with major structural and functional changes. This is due to the production of aluminum as a toxic dust that causes diseases like asthma, chronic bronchitis, chronic pneumonia, chronic obstructive pulmonary disease (COPD), pulmonary fibrosis, pulmonary alveolitis, alveolar proteinosis, pneumoconiosis (e.g., silicosis), and respiratory cancer. Also, workers who work for a long time in potroom suffer from lung function impairment that is called asthma-like syndrome with unidentified pathogenesis. This is called “potroom asthma.” All of these patients experience coughing, shortness of breath, dyspnea on exertion (Doe), phlegm, wheezing, and chest tightness. Chest radiography shows decreased lung fields, irregular opacities, and abnormal patterns of pleura and diaphragm (Figure 1) [19, 62, 63].

Factors that affect brain development include gene expression, axonal transportation, neurotransmitter synthesis, synaptic transmission phosphorylation or dephosphorylation of proteins, protein degradation, and inflammatory responses [64]. Aluminum can change gene expression by reducing the expression of neurofilament, tubulin, transferrin receptor, amyloid precursor protein, and neuron-specific enolase. Also, it alters the expression of RNA polymerase I, oxidative stress, and *β*-APP (*β*-amyloid precursor protein) secretase [64]. Aluminum can bind to the histone-DNA complex and change chromatin conformation and topological DNA. In neurodegenerative brains, the amino acids are mostly in D-configuration (amino acids in normal human brain are in L configuration with a few numbers having D-configuration), particularly D-aspartate (D-Asp) and D-glutamate (D-Glu), which are relatively high in core amyloid plaque, and neurofibrillary tangles (NFTs). D-Asp causes aggregation and fibril formation of the A*β* peptide. Aluminum-D-Asp complex has been shown to modulate DNA topology, and aluminum and A*β* peptides possibly play an important role in B-Z organizational changes in DNA [65]. Aluminum will interfere with cellular function by inhibition of some enzymes such as hexokinase, phosphofructokinase, and glucose-6-phosphate dehydrogenase [64] and has the ability to cause harmful effects on mitochondrial function, antioxidant defense system, and the morphology of the brain. Dysfunction of the mitochondria results in the depletion of the ATP levels, which leads to cytotoxicity and cell death [63]. Aluminum affects phosphorylation or dephosphorylation reactions by inhibiting the activity of protein phosphatase and dephosphorylation of tau and causing the nonenzymatic phosphorylation of tau [64]. It can cause accumulation of amyloid *β* plaques in the brain, tau protein, and degeneration of neurofibrillary tangles in neurons [64]. Neurological manifestations of aluminum poisoning include memory loss, tremor, jerking movements, diminished coordination, weakness in motor movements, lack of curiosity, ataxia, speech disturbances (dyspraxia, dysphasia, stuttering, and probably mutism), myoclonic jerks, and generalized convulsions with status epilepticus, as well as changes in behavior and consciousness levels such as agitation, confusion, myoclonic jerk, grand mal seizures, obtundation, coma, and death [19, 66] (Figure 1).

## 6. Diagnostic Tests

Aluminum is an abundant element in nature, and external sources of this metal may lead to an increase in serum concentrations that result from contamination during collection, processing, or analysis. Another noteworthy point is the clinical finding when blood and urine samples are taken from someone who is exposed to aluminum poisoning [64]. In general, the amount of aluminum in the blood will be less than 10 micrograms per liter or less than 60 micrograms per liter in patients undergoing dialysis [67]. Toxicity occurs at concentrations of more than 100 micrograms per liter [68]. Also, urine aluminum concentrations below 55 *μ*g/g of creatinine are safe for humans. While the level of urinary aluminum to 4 to 6 *μ*mol/L (108 to 162 *μ*g/L) represents a threshold for neurological side effects, urine level of 100 *μ*g per liter is known as the critical concentration and the development of neurological complications [69]. Aluminum poisoning can also be assessed by examining the level of aluminum in hair, nails, and sweat [70].

## 7. Management

After identifying patients exposed to aluminum, they should be avoided from additional contamination through different ways. This action is done with the removal of all environmental or injection and oral sources of this metal. Patients with occupational asthma (potroom) should change their workplaces, and bronchodilators and corticosteroids are prescribed for them. Aluminum chelators are used for the collection of aluminum deposits and reduce the metal load in the body [19, 71]. The standard concentration of aluminum in dialysis water and drinking water is 10 *μ*g/L and 200 *μ*g/L, respectively, in the European countries. Some experts believe that the level of aluminum in the dialysis fluid is less than 2 *μ*g/L, using a double system of reverse osmosis system in their dialysis centers [71, 72]. The use of a double reverse osmosis device allows very low aluminum concentration in dialysate, which has several benefits, including prevention of aluminum toxicity from the dialysate to plasma, as well as removing aluminum from the plasma by hemodialysis [71].

**Figure 2:**
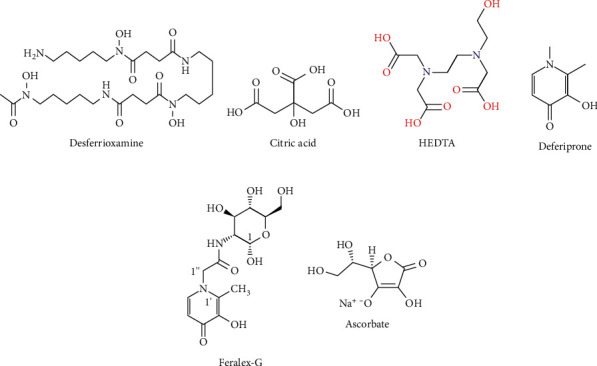
Aluminum chelators for the collection of aluminum deposits and reducing metal load in the body.

Chelation therapy is suggested in acute and chronic cases of poisoning with aluminum salts. Undoubtedly, the only chelator with confirmed useful effects in the case of aluminum poisoning is deferoxamine mesylate (desferrioxamine) (DFO) (Figure 2). The National Kidney Foundation Kidney Disease Outcomes Quality Initiative (NKF KDOQI) recommends the use of DFO when the concentration of aluminum is in the range of 60 to 200 *μ*g/liter in dialysis patients. In acute and chronic cases and minimizing anticipated encephalopathy, DFO is prescribed. Chronic dialysis patients will receive a dose of 5 mg/kg of DFO (or sometimes a lower dose) for more than an hour before the scheduled dialysis session. It is also prescribed in acute conditions at a higher dose of 15 mg/kg/day. Deferoxamine binds to aluminum and forms aluminoxamine chelate which is excreted by urine or hemodialysis. The duration of use differs in dementia, osteomalacia, and encephalopathy [19, 73, 74].

Calcium disodium ethylenediaminetetraacetic acid (CaNa2EDTA or EDTA) is one of the chelators that has proven its effectiveness in reducing aluminum intoxication significantly. Intravenously infusion of 2 g/10 ml EDTA diluted 500 ml physiological saline for about 2 hours. Treatment was given once a week, lasting for ten weeks in one study, and in another, the duration continued as once every two weeks, which lasted for six or twelve months. The results showed that improvement of the neurological symptoms in these patients was in parallel with reduction of aluminum levels in their urine samples [75, 76].

Ordog claimed that aluminum factory workers who have been suffering from aluminum poisoning had complications such as sinus infections, chronic fatigue, lack of sensation and movement, ataxia, vertigo, memory loss, and chronic pain. They were treated with meso-2,3-dimercaptosuccinic acid (Succimer, DMSA) 900 mg twice daily for 19 days. He pointed out that all patients had a positive response to treatment. He reported that DSMA is helpful as a clinical chelator in the case of aluminum poisoning [77].

### 7.1. Combine Therapy

Chelator combinations that have the highest efficiency in removing aluminum include ascorbate (AS) (vitamin C), deferoxamine (DFO), which is recognized by the FDA as a well-known drug, and Feralex-G (FG) (a new chelator used in the mouth). This compound is very effective in removing aluminum from different cells of the human brain [78, 79] (Figure 2).

Another chelator that resembles EDTA is N-(2-hydroxyethyl) ethylenediaminetriacetic acid (HEDTA). It is reported to be a potential chelator for aluminum. Besides, propolis is another compound that is a potential antioxidant and antilipid peroxidation against oxidative stress. Propolis is a natural product that is collected from honey bees from buds, exudates of trees, and other components of the plants and is mixed with salivated secretions and wax in the hive. Combination treatment with HEDTA and propolis can improve the damaged cellular membrane and organ functioning. The activity of acetylcholinesterase notably decreases in the front, middle, and back of the brain after exposure to aluminum, which may be due to intervention with the synthesis of AChE or prevention choline uptake by synaptosomes. The simultaneous administration of HEDTA and propolis regulates the activity of the AChE, thus providing nerve protection, reducing oxidative stress, and preserving cell membrane [80].

As stated, aluminum reduces the activity of acetylcholinesterase and also exposure to aluminum and significantly reduces the activity of gamma-aminolevulinic acid dehydratase (ALAD) in blood and gamma-aminolevulinic acid synthesis (ALAS) in brain. Treatment with HEDTA and selenium (Se) reduces the accumulation of aluminum and regulates cellular signaling, neurotransmission, and biophysical changes in the membrane [81]. DFO cannot enter the brain, but HEDTA can pass through the blood-brain barrier (BBB) and lead to a moderate reduction in the concentration of aluminum in the brain. For optimum outcome in the treatment, the combination of citric acid (CA) (which decreases oxidative stress and removes aluminum from the storage and redistribute in the plasma) with HEDTA is used [82] (Figure 2).

Among chelating agents tested in the experimental animal model, one of the most useful and successful agents was 1-alkyl-3-hydroxy-2-methylpyrid-4-one (deferiprone (DFP)) or ferriprox (Figure 2), which can be used with the combination 1,2-dimethyl-3-hydroxypyrid-4-one (L1) [83]. The combination of both DFO and L1 increases the elimination of aluminum and iron simultaneously. Of course, the investigation has shown that the L1 does not increase the effect of the DFO on aluminum, and the addition of DFO to L1 will not increase the effect of L1 on iron excretion [84].

Currently, for effective chelating agents to form complexes with high stability, selectivity, lipophilicity, and bioavailability, some of the derivatives of kojic acid were synthesized, which showed high efficacy in the elimination of aluminum and iron ions. Of course, further research on the toxicology and pharmacology of these new ligands is recommended to improve the properties of these chelators [85].

## Figures and Tables

**Table 1:** Mechanism of aluminum poisoning and its clinical presentations.

Organs	Kidney	Brain	Parathyroid and musculoskeletal	Bone marrow and hematopoiesis	Liver	Lungs
Mechanism of aluminum poisoning	Increasing oxidative stress and lipid peroxidation as well as oxidative stress to DNA and proteins Decreasing GSH content and GSH-Px, GST, and CAT activities Changing renal-tubular p-aminohippuric acid transport, renal-tubular phosphate reabsorption, and sodium water balance impairment Inhibiting Na+/K + ATPase action Increasing free intracellular Fe^+2^ resulting in oxidative stress	ROS generation leads to lipid peroxidation, MMP inhibition, ATP reduction, neurotransmitter dysfunction, and neural death Preventing DNA and RNA formation Preventing DNA repair Inhibiting PP2A activity resulting in tau and neurofilament proteins hyperphosphorylation Increasing biosynthesis of the transferrin receptor prevention of ferritin production increase iron in cytoplasmic pools of neurons leading to oxidative stress Corticoneuronal apoptosis through SAPK/JNK pathway Increasing NF-ķ*β* activation Increasing p53 and BAX resulting in apoptosis Reducing gene expression of neurofilament, tubulin, transferrin receptor, amyloid precursor protein, and neuron-specific enolase Altering expression of RNA polymerase I, oxidative stress, and *β*-APP secretase Interfering with cellular function Amyloid *β* accumulation in the brain and theophyllation of tau Increasing free intracellular Fe^+2^ resulting in oxidative stress	Reducing parathyroid response to hypocalcemia Inhibiting remodeling and decreasing the number of osteocytes and osteoblasts at higher doses	Inhibiting hemoglobin synthesis	Degeneration in RER that results in a reduction in protein synthesis and change in Ca^+2^ levels Swelling of liver cells mitochondria and permeability change in mitochondria membrane Increase ALT and MDA activity up to 4 times Increase proinflammatory cytokines such as TNF-*α* up to seven times Increase free intracellular Fe^+2^ that leads to oxidative stress and anaerobic respiration and decreases ATP production Inhibits Na+/K + ATPase action	Increased PMN influx Minimal interstitial inflammation Type II cell hyperplasia Decrease in alveolar lavageable macrophages
Pathophysiology and clinical manifestations	Reduction in glomerular filtration Changing serum levels of urea, creatinine, and uric acid Nephrotic syndrome Acute renal glomerulonephritis	Memory loss Tremor Jerking movements Diminished coordination Weakness motor movement Lack of curiosity Ataxia Speech disturbances Myoclonic jerks Generalized convulsions Agitation and confusion Grand mal seizures Obtundation Coma Death	Hypothyroidism osteoporosis osteomalacia Decreased bone formation Nonhealing fractures Erosive arthropathy with cysts Spondyloarthropathy Calcium deposition in soft tissue Amyloidosis Chondrocalcinosis	Anisocytosis Poikilocytosis Leptocyte formation Acanthocyte formation Echinocyte formation Stomatocyte formation Target cell formation Microcytic hypochromic anemia	Obesity Type 2 diabetes liver steatosis	Asthma Chronic bronchitis Chronic pneumonia Pulmonary alveolitis COPD Pulmonary fibrosis Alveolar proteinosis Pneumoconiosis Potroom asthma

GSH: glutathione, GSH-Px: glutathione peroxidase, GST: glutathione S-transferase, CAT: catalase, ROS: reactive oxygen species, MMP: mitochondrial membrane potential, PP2A: protein phosphatase 2A, SAPK/JNK: stress-activated protein kinase or c-Jun N-terminal kinase, BAX: B-cell lymphoma- Associated X, *β*-APP: *β*-amyloid precursor protein, RER: rough endoplasmic reticulum, ALT: alanine aminotransferase, MDA: malondialdehyde, PMN: polymorphonuclear leukocyte, and COPD: chronic obstructive pulmonary disease.

**Table 2:** Main elements in the alloys, heat and nonheat treatable alloys, and main groups of cast aluminum alloys.

Alloy series	Main elements in the alloy	Heat and nonheat treatable alloys
1xxx	With a purity of 99% or higher aluminum	
2xxx	Copper^*∗*^	Heat treatable alloy
3xxx	Manganese	Nonheat treatable alloy
4xxx	Silicon^*∗*^	Nonheat treatable alloy
5xxx	Magnesium^*∗*^	Nonheat treatable alloy
6xxx	Magnesium and silicon	Heat treatable alloy
7xxx	Zinc^*∗*^	Heat treatable alloy
8xxx	Other elements	

^*∗*^Cast aluminum alloys.

## Data Availability

The data used to support this study are available from the corresponding author upon request.

## Supplementary Materials

Supplementary Materials Mechanism of aluminum poisoning. Al produces reactive oxygen species, resulting in lipid peroxidation and oxidative damage to proteins and DNA. Al poisoning also increases free Fe^2+^ resulting in further oxidative stress. Al toxicity also reduces the content and activity of antioxidant enzymes, inhibits Na+/K + ATPase action, and alters cell membrane permeability. An increase in cytoplasmic calcium, p53, and BAX proteins due to Al poisoning leads to apoptosis.
