# Supplementary figures and images for: Aluminum Poisoning with Emphasis on Its Mechanism and Treatment of Intoxication
Source: Emerg Med Int. 2022 Jan 11;2022:1480553. doi: 10.1155/2022/1480553 (PMC8767391; doi:10.1155/2022/1480553)

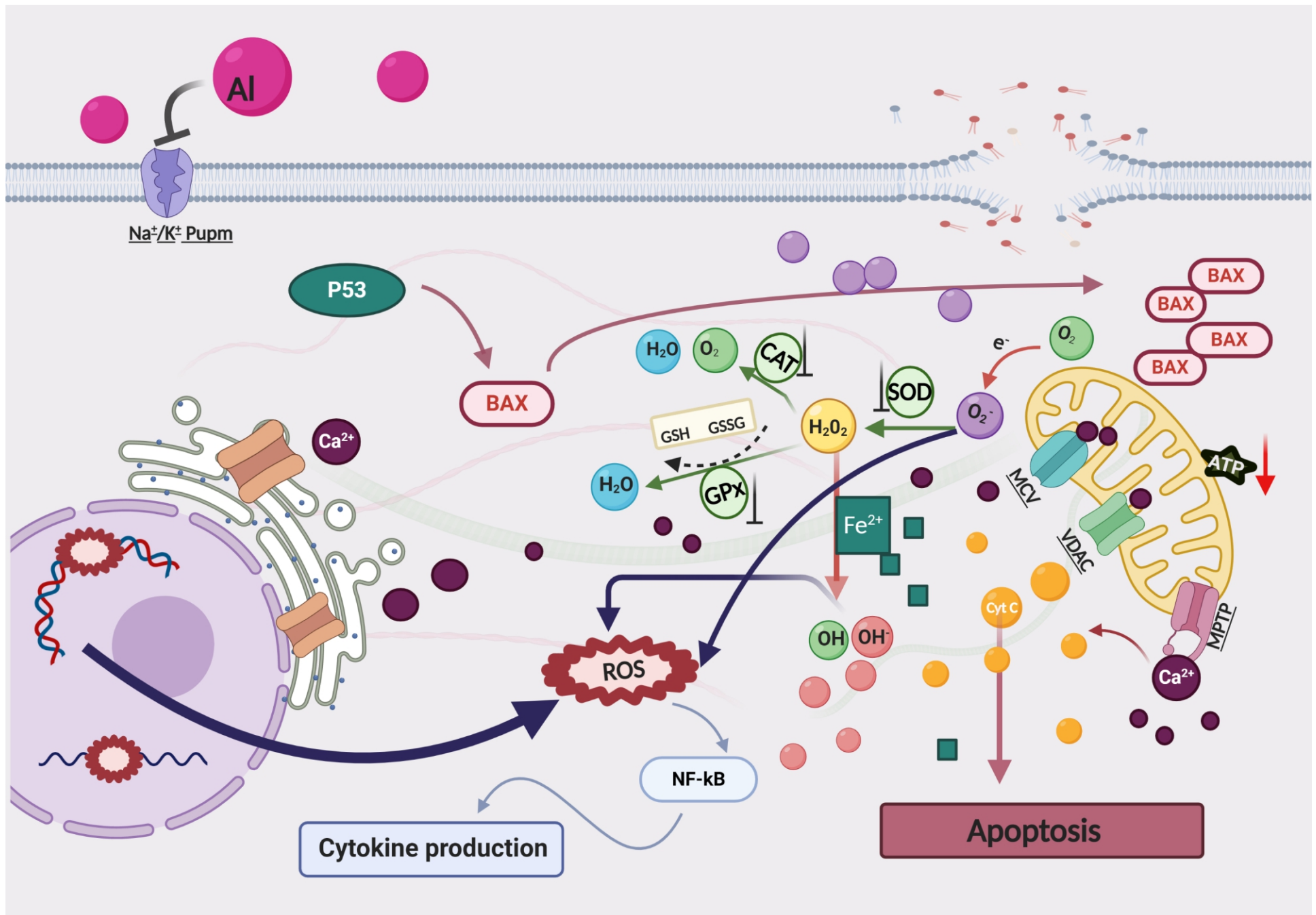

Supplement: Supplementary Materials — Mechanism of aluminum poisoning. Al produces reactive oxygen species, resulting in lipid peroxidation and oxidative damage to proteins and DNA. Al poisoning also increases free Fe2+ resulting in further oxidative stress. Al toxicity also reduces the content and activity of antioxidant enzymes, inhibits Na+/K + ATPase action, and alters cell membrane permeability. An increase in cytoplasmic calcium, p53, and BAX proteins due to Al poisoning leads to apoptosis. [file 1480553.f1.pdf]
